# *In vitro* and *in vivo* efficacy study of cefepime, doripenem, tigecycline, and tetracycline against extended-spectrum beta-lactamases *Escherichia coli* in chickens

**DOI:** 10.14202/vetworld.2020.446-451

**Published:** 2020-03-11

**Authors:** Yaser Hamadeh Tarazi, Ehab A. Abu-Basha, Zuhair Bani Ismail, Rawan A. Tailony

**Affiliations:** 1 Department of Basic Medical Veterinary Sciences, Faculty of Veterinary Medicine, Jordan University of Science and Technology, Irbid, Jordan; 2 Department of Veterinary Clinical Sciences, Faculty of Veterinary Medicine, Jordan University of Science and Technology, Irbid, Jordan

**Keywords:** antimicrobial resistance, chickens, colibacillosis, *Escherichia coli*, multidrug-resistant-extended-spectrum beta-lactamases

## Abstract

**Background and Aim:**

At present, there are no data about the efficacy of some recent antibiotics on *Escherichia coli* in broiler chickens in the study area. This study was designed to evaluate the *in vitro* and *in vivo* efficacy of cefepime, doripenem, tigecycline, and tetracycline against multidrug-resistant-extended-spectrum beta-lactamases (MDR-ESBLs) producing *E. coli* in broiler chicks.

**Materials and Methods:**

A total of 34 MDR-ESBLs *E. coli* isolates were used in this study. *In vitro* evaluation of the antibacterial efficacy of cefepime, doripenem, tigecycline, and tetracycline were performed using disk diffusion and minimum inhibitory concentration (MIC) assays. *In vivo* evaluation of the efficacy of the antibiotics was perfumed using 180, 2-week-old chicks challenged with MDR-ESBL-producing *E. coli* strain O78. Chicks were divided into six groups (30 chicks each) according to the treatment regimen. Treatment was administered to chicks in Groups 3-6 intravenously, twice per day for 1 week using one antibiotic per group at concentration 10 times the determined MIC. Chicks in the positive control (Group 1) were challenged and received 0.2 ml of sterile Tryptone Soy Broth (TSB), while those in the negative control (Group 2) were not challenged and received 0.2 ml of sterile TSB. The severity of clinical signs, gross lesions, and mortality rate was scored and compared between groups.

**Results:**

All *E. coli* isolates were sensitive to doripenem and tigecycline, while 88% were sensitive to cefepime and only 23% were sensitive to tetracycline. *In vivo* antibiotic efficacy evaluation in challenged chicks revealed a significant reduction in the severity of clinical signs, gross lesions, and mortality (3%) in chicks treated with cefepime compared to non-treated chicks (55%). There was no significant effect on the severity of clinical signs, gross lesions, and mortality in chicks treated with doripenem, tigecycline, and tetracycline compared to non-treated chicks. The mortality rates of chicks treated with doripenem, tigecycline, and tetracycline were 57%, 50%, and 90%, respectively.

**Conclusion:**

The results of this study indicate that most MDR-ESBLs producing *E. coli* isolates were sensitive to doripenem, tigecycline, and cefepime. However, *in vivo* study indicated that only cefepime was effective and resulted in a significant reduction in clinical signs, gross lesions, and mortality in infected chicks. Therefore, cefepime could be used to treat naturally infected chickens with MDR-ESBLs producing strains of *E. coli*.

## Introduction

The emergence of infectious diseases caused by multidrug-resistant (MDR) pathogens has become a serious global health problem with significant increase in morbidity and mortality [1,2]. In recent years, many life-threatening diseases caused by methicillin-resistant *Staphylococcus aureus*, extended-spectrum beta-lactamases (ESBLs) producing *Escherichia coli*, and MDR strains of *Salmonella* spp., *Klebsiella* spp., and *Pseudomonas aeruginosa* have become prevalent causing significant suffering in both humans and animals [3,4].

MDR *E. coli* is of particular concern because it is the most common Gram-negative opportunistic pathogen in humans and animals, causing urinary tract infections, diarrhea, and community- and hospital-acquired bacteremia [5]. ESBLs production by *E. coli* is responsible for resistance of the bacteria against a multitude of antimicrobial agents, mainly members of the cephalosporin and penicillin families [6]. Beta-lactamase hydrolyzes the β-lactam ring in these antibiotics rendering them ineffective [7]. ESBLs producing *E. coli* is considered a public health threat due to the risk of plasmid transfer of the ESBLs resistance genes to human pathogens from *E. coli* strains of animal origins [6,7].

The risks of antibiotic resistance spread among human and animal pathogens are seriously concerning to global health officials [8]. Although this process cannot be completely prevented, it can be slowed down through implementing strict antibiotic use regulations that ensure the judicious use of available antibiotics both in humans and animals and the development of new and effective antimicrobial agents [9-17].

We are running out of options to treat bacterial diseases due to widespread of antimicrobial resistance in poultry, the use of recent antibiotics could be a good option for treating bacterial diseases caused by MDR-ESBLs producing *E. coli* in chickens [18- 21].

This study was designed to investigate the *in vitro* and *in vivo* efficacy of cefepime, doripenem, tigecycline, and tetracycline against MDR-ESBLs *E. coli* isolates in chicks.

## Materials and Methods

### Ethical approval

This study was reviewed and approved by the Institutional Animal Care and Use in research at Jordan University of Science and Technology (ACUC, Project # 2019/48).

### Bacterial culture and preparation

A total of 34 MDR-ESBLs producing *E. coli* isolates were used in this study. The bacteria were isolated from clinical samples obtained from naturally infected chickens and accumulated in Microbiology Research Laboratory from the previous study. *E. coli* isolates and serotype O78 used in the challenge assay in this study showed MDR pattern against >11 antimicrobial agents and was accumulated in Microbiology Research Laboratory, Faculty of Veterinary Medicine, Irbid-Jordan from the previous study. The isolates considered as ESBLs producer because they are resistant to penicillin, cephalosporin, and aztreonam and confirmed by double-disk synergy test as demonstrated in the materials and methods in this study.

The isolates were cultured on MacConkey and eosin methylene blue agar plates, incubated at 37°C for 24 h. Bacterial identification was carried out based on colony morphology, Gram’s stain characteristics, and biochemical characteristics including oxidase test and indole, methyl red, Voges–Proskauer, and citrate test[22].

### Antimicrobial agent’s preparation

Raw materials of antibiotics of doripenem, tigecycline, and tetracycline were provided by Cayman Chemical Company (Michigan, USA) and cefepime was provided by Demo S.A. Pharmaceutical (Votanikos, Greece). Doripenem and cefepime were suspended in saline while tigecycline and tetracycline were suspended in distilled water. The antibiotic disks of tigecycline (15 µg), doripenem (10 µg), oxacillin (1 µg), and cefoxitin (30 µg) were provided by Oxoid (Hampshire, UK), while cefepime (30 µg) and tetracycline (30 µg) were provided by Bioanalyse (Ankara, Turkey). The antibiotic E-strips of tigecycline (0.016-256 µg/ml), tetracycline (0.016-256 µg/ml), cefepime (0.016-256 µg/ml), oxacillin (0.016-256 µg/ml), cefoxitin (0.016-256 µg/ml), and doripenem (0.002-32 µg/ml) were provided by HiMedia (Mumbai, India).

### Antimicrobial sensitivity test

The antimicrobial sensitivity test was performed using the Kirby–Bauer disk diffusion method on Mueller-Hinton agar (Oxoid, UK). The fresh culture of ESBLs *E. coli* was diluted in normal saline to 0.5 McFarland standards (~1×10^8^ CFU/ml). From this suspension, the surface of Mueller-Hinton agar plates was swabbed in four directions and the plates were left to dry and then the antibiotic discs were added. The plates were then incubated at 37°C for 24 h and the inhibition zones were recorded[23].

### Minimum inhibitory concentration (MIC)

The MIC was performed using E-strip (Epsilometer; BioMérieux, France) according to the manufacturer’s recommendations. The inoculum was prepared in Mueller-Hinton agar broth (Oxoid, UK) diluted to a 0.5 McFarland standard. Then, the antibiotic strip was placed on the surface of Mueller-Hinton agar plate and incubated at 37°C for 24 h. The MIC values were recorded at the points of intersection of the inhibition zones with the graded strips[23].

### ESBLs production confirmation by E. coli

The production of ESBLs by the isolated *E. coli* strains used in this study was confirmed by double-disk synergy test[24]. All resistant strains against third-generation cephalosporin were used to detect ESBL production. Briefly, a disk containing cefotaxime (30 μg) was placed at 15 mm away from a centrally placed disk containing amoxicillin-clavulanic acid (20 μg/10 μg). The plates were then incubated at 37°C for 24 h. The isolate was considered ESBLs producer if it showed a distinctive inhibition zone potentiated by amoxicillin/clavulanic acid disk[24,25].

### E. coli pathogenicity assay

The pathogenicity test aimed to determine which of the isolates is capable of causing the highest mortality to be used in the subsequent chick challenge test. Three O78 *E. coli* isolates were among the 34 MDR-ESBLs producing *E. coli* were used to evaluate pathogenicity by challenging 10, 2-week-old chicks by intrathoracic inoculation of the bacteria. Each chick received approximately 0.2 ml inoculum (1×10^9^ CFU *E. coli*/ml) was injected into the left caudal thoracic air sac[26,27]. The chicks were monitored daily for 7 days and the mortality rate was calculated.

### E. coli challenge assay

One hundred and eighty, 2-week-old chicks (Hubbard Classical) were obtained from a local company (Amman, Jordan) and maintained in the animal house, Faculty of Veterinary Medicine, Jordan University of Science and Technology. Chicks were housed in cages at room temperature of 27-29°C. Freshwater and a commercial broiler chick feed were provided *ad libitum*. The feed was free of antimicrobial and anticoccidial drugs. Birds were provided with a continuous lighting pattern for 24 h during the experiment.

The chicks were divided into six groups (30 chicks each). In Group 1, chicks were infected and not treated (positive control); while in Group 2, the chicks were not infected and not treated control. In Groups 3-6, chicks were infected and treated with doripenem, cefepime, tigecycline, and tetracycline, respectively[28]. The antibiotics were administered at a dose rate equivalent to 10 times the MIC, intravenously in the wing vein twice daily for a week. Chicks in the control groups received 0.2 ml of sterile Tryptone Soy Broth intravenously in a manner similar to the chicks receiving antibiotics treatment.

Before inoculation, chicks were allowed to acclimatize in their environment for 1 week. The chicks were monitored daily during this period to determine their health status. After the challenge, chicks were monitored daily for expected clinical signs including depression, reluctance to move, gasping, and difficult breathing. The number of dead chicks was recorded daily for 7 days. On day 8 after inoculation, all chicks were humanely euthanized by cervical dislocation and a thorough necropsy was performed[29]. Gross lesions involving the major abdominal and thoracic organs, including the liver, heart, and air sacs, were reported and scored according to previously published methods[30].

### Statistical analysis

The mean ranks of gross lesions involving different body organs were determined using Kruskal–Wallis test. Statistical differences in the scores of gross lesions in different body organs between different treatment groups were analyzed using Mann–Whitney U-test. Statistical analysis was performed using the SPSS software version 24 (IBM, NY, USA). Statistical difference was considered statistically significant at p≤0.05.

## Results

### Antimicrobial sensitivity test

**Figure-1:**
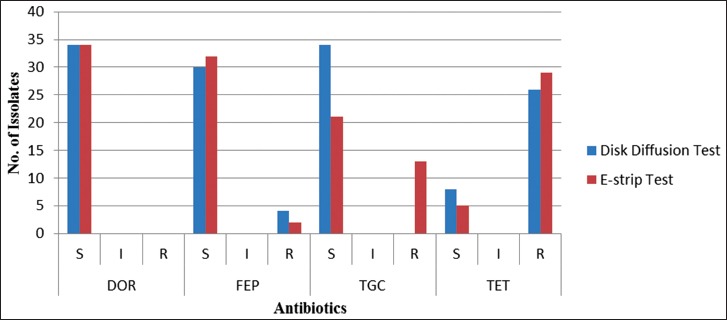
Antibiotic resistance profile of 34 isolates of extended-spectrum beta-lactamases producing *Escherichia coli* against cefepime, doripenem, tigecycline, and tetracycline. DOR=Doripenem, FEP=Cefepime, TGC=Tigecycline, TET=Tetracycline, S=Susceptible, I= Intermediate, R= Resistant.

The results of the disk diffusion sensitivity test and MIC of cefepime, doripenem, tigecycline, and tetracycline against MDR-ESBLs producing *E. coli* are presented in Figure-1. According to the disk diffusion test, all *E. coli* isolates were sensitive to doripenem and tigecycline while 88% were sensitive to cefepime and only 23% were sensitive to tetracycline. The MIC results showed that most *E. coli* isolates were more sensitive to doripenem and cefepime antibiotics.

The three MDR-ESBLs producing *E. coli* isolate used in pathogenicity assay were of serotype O78 isolated from naturally infected broiler chickens from the previous study. The isolates were sensitive to cefepime, doripenem, and tigecycline and resistant to tetracycline.

### Clinical signs and postmortem lesions

**Figure-2:**
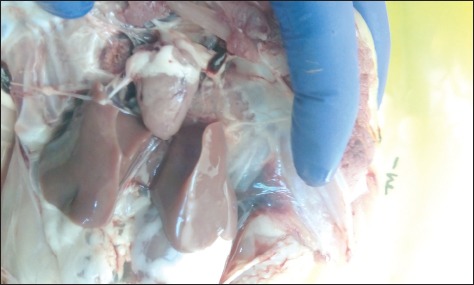
No gross lesions could be observed in non-challenged control chicks.

**Figure-3:**
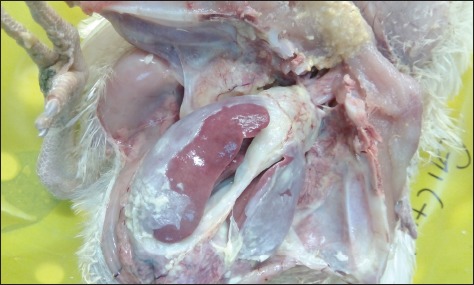
Infected chick with multidrug-resistant-extended-spectrum beta-lactamases producing *Escherichia coli* with no treatment administered (positive control) showing severe fibrinous formation on the heart, liver, and air sacs.

**Figure-4:**
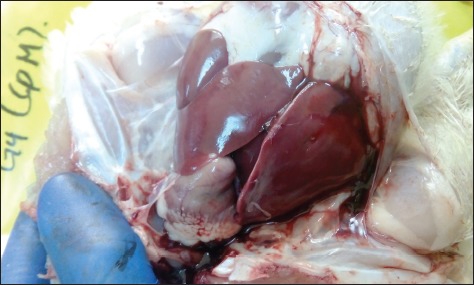
Infected chick with multidrug-resistant-extended-spectrum beta-lactamases producing *Escherichia coli* treated with cefepime showing slightly congested liver with no to little accumulation of fibrinous materials on the heart, liver, and air sacs.

Chicks in the negative control group showed no apparent clinical signs or postmortem lesions (Figure-2). In the positive control group, the chicks showed severe depression, fatigue, reluctance to move, and respiratory signs with a mortality rate of 55%. Gross lesions were consistent with the formation of fibrinous material on the heart, liver, and air sacs (Figure-3). In Group 4 (infected and treated with cefepime), chicks showed no apparent clinical signs and only one chick died out of 30 (3% mortality rate). Gross lesions were slight liver congestion and little fibrinous material accumulation on the heart with cloudy air sac (Figure-4). In Group 3 (infected and treated with doripenem), the clinical signs were similar to that of the positive control group. Seventeen chicks died in this group with a mortality rate of 57%. Gross lesions were severe accumulation of fibrinous material on the heart, liver, and air sacs. In addition, surviving chicks also showed severe postmortem lesions affecting the heart, liver, and air sacs. In Group 5 (infected and treated with tigecycline), clinical signs were almost similar to that of the positive control group with 15 dead chicks (50% mortality rate). The gross lesions in the dead and surviving chicks were similar to those observed in the positive control group. In Group 6 (infected and treated with tetracycline), clinical signs and postmortem lesions were similar to those observed in the positive control group with a mortality rate of 90%.

In general, the differences between the groups were significant (p≤0.05) when the comparison was made between treated groups and non-treated chicks (Table-1). However, statistical analysis of the lesion scores of visceral organs revealed that chicks in Group 4 (treated with cefepime) exhibited the lowest mean rank compared to all other groups.

**Table-1:** Mean ranks of gross lesions in different body organs in chicks infected with multidrug-resistant ESBLs producing *E. coli*.

Organs	Groups					

	1	2	2	4	5	6
Liver	144^a^	20^ăe^	105^ăē^	76^ăēc^	119^ăč^	132^a^
Heart	145^a^	23^ăe^	103^ăē^	74^ăēc^	108^ăč^	140^a^
Air-sacs	154^a^	27^ăe^	95^ăē^	74^ăēc^	104^ăč^	129^ă^

^aă^Indicate significant difference at p≤0.05. ^eē^Indicate significant difference at p≤0.05. ^cč^Indicate significant difference at p≤0.05. *E. coli=Escherichia coli*, MDR-ESBL=Multidrug-resistant extended-spectrum beta-lactamases

## Discussion

Treatments of many infectious diseases have become a great challenge due to the emergence of multidrug antibiotic resistance among pathogenic microorganisms[31]. Indeed, MDR microorganisms are now widespread in the environment and are posing serious threats to public health worldwide[32]. Commercial development of new classes of antibiotics has diminished over the past 15 years and few pharmaceutical companies remain active, which indicates an urgent need for new medications to overcome the rising problem of MDR microorganisms[32]. β-lactams remain the mainstay antibiotic therapeutic agents against many bacterial infections [24]. Unfortunately, injudicious use of these antimicrobial agents over the years rendered then ineffective [9,33]. Therefore, in this study, a new generation of antibiotics has been chosen to evaluate their efficacy against MDR-ESBLs producing *E. coli* in chickens.

Avian pathogenic *E. coli* (APEC) is associated with huge economic losses to the poultry industry due to high prevalence rates of multidrug resistance among this bacterium [34]. It has been demonstrated that most APEC isolated from broilers are resistant to sulfamethoxazole-trimethoprim, florfenicol, amoxicillin, doxycycline, spectinomycin, tetracycline, and erythromycin [34].

In this study, MDR-ESBLs producing *E. coli* were found highly sensitive to doripenem and cefepime showing the largest inhibitory zone among all tested antibiotics. In addition, results of the MIC breakpoints revealed that this microorganism is highly susceptible to both antibiotics. These results are in total agreement with previously published data [25,35]. In addition, six of the tested *E. coli* isolates (18%) were found resistant to cefepime which is similar to the previous findings reported previously by Mansouri *et al*. [36]. Thirteen *E. coli* isolates were found resistant to tigecycline. This also was similar to the results obtained by Wang *et al*. [37]. Twenty-three *E. coli* isolates were found sensitive to tetracycline, which also similar to the results obtained by Kabiru *et al*. [28].

In this study, the challenge test aimed to determine the effectiveness of these antimicrobial agents on infections caused by MDR-ESBLs producing *E. coli* in broiler chicks. Induction of colisepticemia by inoculating *E. coli* (O78) directly to the air sac is an effective approach in producing clinical colibacillosis and colisepticemia in broiler chicks [38]. Colisepticemia usually occurs within 3-12 h after inoculation, through bacterial passage across the air capillary walls [38]. The pathogenicity of *E. coli* strain used in the challenge test in this study was previously determined [30]. The challenged bacteria were MDR-ESBLs producing *E. coli* serotype O78 which possess several virulence-associated genes obtained from broiler chickens suffering from chronic respiratory disease[39]. In the challenged chicks, the clinical signs and gross lesions in non-treated chicks were consistent with severe colibacillosis. The mortality rate was up to 55% in this group. In chicks that were treated using different antibiotics, the clinical signs, gross lesions, and mortality rates were variable compared to the control groups. For example, in doripenem- and tigecycline-treated chicks, the mortality rates were 57 and 50%, respectively. This indicates that doripenem and tigecycline were not effective for the treatment of infection caused by this strain of *E. coli*. Although, both of these antibiotics showed 100% susceptibility against *E. coli* in the *in vitro* study. The difference between *in vitro* susceptibility of antibiotics and *in vivo* efficacy is commonly encountered in clinical practice. This can only be explained by performing specialized biokinetic studies. However, similar results have been obtained previously by Samonis *et al*. [40].

In challenged chicks treated by tetracycline, the mortality rate was 90%. In the *in vitro* susceptibility test, 77% of *E. coli* isolates were resistant to tetracycline *in vitro*. This means that tetracycline is not an effective drug for the treatment of colibacillosis in broiler chicks. The high resistant profile of *E. coli* against tetracycline is similar to the results of Al-Bahry *et al*. [41]. In challenged chicks treated with cefepime, the mortality rate was only 3%. The *in vitro* susceptibility test demonstrated that 88% of the tested *E. coli* isolates were susceptible to cefepime. In fact, cefepime-treated chicks showed obvious improvement in clinical signs and reduction in the scores of gross lesions and a significant (p*≤*0.05) reduction in mortality rate in comparison to those in the untreated control group. These results are similar to previously reported findings in broiler chickens [42].

## Conclusion

The results obtained from this study indicated that cefepime is an effective antimicrobial agent against infections caused by MDR-ESBLs producing *E. coli* in broiler chicks. Therefore, this antibiotic could be used in the treatment of naturally occurring colibacillosis in chickens.

## Authors’ Contributions

YHT designed the project protocol, supervised the whole work, and wrote the manuscript. EAA and ZBI involved in the manuscript writing and supervised the pathogenicity and challenge assay. RAT did the laboratory works. All authors read and approved the final manuscript.
